# Evaluation and optimization of microbial DNA extraction from fecal samples of wild Antarctic bird species

**DOI:** 10.1080/20008686.2017.1386536

**Published:** 2017-10-26

**Authors:** Per Eriksson, Evangelos Mourkas, Daniel González-Acuna, Björn Olsen, Patrik Ellström

**Affiliations:** ^a^ Zoonosis Science Center, Department of Medical Sciences, Uppsala University, Uppsala, Sweden; ^b^ Zoonosis Science Center, Department of Medical Biochemistry and Microbiology, Uppsala University, Uppsala, Sweden; ^c^ The Milner Centre for Evolution, Department of Biology and Biochemistry, University of Bath, Bath, UK; ^d^ Facultad de Ciencias Veterinarias, Universidad de Concepción, Chillán, Chile

**Keywords:** Antarctica, Aves, DNA extraction, feces, method evaluation and scatology

## Abstract

**Introduction**: Advances in the development of nucleic acid-based methods have dramatically facilitated studies of host–microbial interactions. Fecal DNA analysis can provide information about the host’s microbiota and gastrointestinal pathogen burden. Numerous studies have been conducted in mammals, yet birds are less well studied. Avian fecal DNA extraction has proved challenging, partly due to the mixture of fecal and urinary excretions and the deficiency of optimized protocols. This study presents an evaluation of the performance in avian fecal DNA extraction of six commercial kits from different bird species, focusing on penguins.

**Material and methods**: Six DNA extraction kits were first tested according to the manufacturers’ instructions using mallard feces. The kit giving the highest DNA yield was selected for further optimization and evaluation using Antarctic bird feces.

**Results**: Penguin feces constitute a challenging sample type: most of the DNA extraction kits failed to yield acceptable amounts of DNA. The QIAamp cador Pathogen kit (Qiagen) performed the best in the initial investigation. Further optimization of the protocol resulted in good yields of high-quality DNA from seven bird species of different avian orders.

**Conclusion**: This study presents an optimized approach to DNA extraction from challenging avian fecal samples.

## Introduction

The interest in microbial ecosystems of humans and other animals has increased tremendously in recent years. Many of these studies have focused on the microbiota of the gut. Analysis of the fecal microbiota can provide information about, for example, the host’s metabolism, health status and/or dietary intake [1–3].

Most studies have been focusing on feces of human or other mammalian origin, but the number of studies on other vertebrates is increasing [2]. Gut microbiota analysis may be regarded as a twenty-first-century science, but the field was pioneered already in the late nineteenth century [4]. Some of the first attempts to determine the microbiota of animals living in the polar regions were made by Levin, who investigated the gut microbiota of various animals from polar bears to sea ducks [5]. Due to culture-dependent analysis techniques, Levin struggled to identify gut microorganisms and thus concluded that the gut of most Arctic animals was sterile. In retrospect, this can be viewed as an example highlighting the crucial importance of using appropriate sample storage and analysis techniques to come to the most accurate conclusion. Investigations of the Antarctic bird microbiota based on culture-dependent methods continued during the twentieth century [6]. However, DNA sequencing launched the new field of culture-independent analysis of the microbial community. The affordable cost nowadays of performing tests such as clonal libraries [7,8], qPCR [9], microarrays [10], terminal restriction fragment length polymorphism [11–14] and next-generation sequencing technologies [11,14–17] opened access to new approaches in characterizing microbial communities in the gastrointestinal tract of various animal species.

Another area where microbial culture has largely been replaced by nucleic acid-based analysis techniques is infection biology, where screening for pathogens in fecal samples and monitoring the dynamics of experimental infection in the gut is performed by tests such as PCR [18,19]. Hence, the challenges of microbial culture are bypassed by culture-independent techniques, but such techniques require pure DNA and the issue has now turned towards extraction and purification of nucleic acids [20]. Indeed, DNA extraction from feces has proven to be challenging. Today there are many different DNA isolation kits available on the market, some being marketed as designed especially for DNA extraction from feces. Most of the studies evaluating DNA extraction methods have been performed on human feces [21–26], with only a few focusing on other animal species [20,27–31]. However, feces are a very diverge sample type and the composition of the fecal material varies greatly, e.g. between mammals and birds [32].

In contrast to mammals, birds have important differences in the physiology of their digestive tract [32]. Special organs of the avian digestive tract include the crop, gizzard and the cloaca. Food can be temporarily stored in the crop and is later mechanically degraded in the gizzard. After being further processed in the lower intestine, the digest is mixed in the cloaca with urinary material and deposited as a moist semiliquid macerate. This makes DNA extraction from avian feces challenging, due to the high content of e.g. uric acid [20,32]. Although extractions of DNA from bird feces have been described earlier [20,33–35], extraction methods have been difficult to reproduce conclusively. Hence, it is of interest to optimize an extraction protocol for avian feces with a convenient yield.

There are a number of different approaches to DNA extraction from feces, ranging from traditional liquid-liquid phase separation (e.g. phenol-chloroform extraction), via column based liquid-solid phase separation (e.g. spin columns) to bead-based liquid-solid phase separation, where the latter easily can be automated [36]. Regardless of the method used, DNA extraction can be divided into three main steps: isolation, washing and elution. Depending on the composition of the sample, it may need to undergo a pretreatment process before entering the extraction [37]. Such pretreatment often includes some kind of degradation of the tissue/sample material, e.g. cell lysis. Cell lysis is usually obtained via chemical, mechanical or enzymatic treatment or a combination thereof. Lysing the crude sample and extracting the DNA may be a trade-off between yielding pure DNA and breaking down and/or losing the material of interest. DNA extraction is thus an area of optimization highly dependent on the sample source itself [20]. The aim of this study was to evaluate commercially available DNA extraction kits and to further optimize a methodology for microbial DNA extraction from feces of different bird species.

## Materials and methods

### Outline

In the initial investigation six different DNA extraction kits were tested using mallard feces. Comparisons were made using at least two sample replicates. The DNA extraction kit used in most of the studies of feces from penguins and other birds in Antarctic regions is the QIAamp DNA Stool Mini kit (Qiagen AB, Sollentuna, Sweden) [34,38–40]. Therefore, this kit was included in the current study, as well as five other kits widely used for fecal DNA extraction. The six DNA extraction kits evaluated were the following: PowerSoil DNA Isolation Kit (MO BIO Laboratories Inc., Carlsbad, CA, USA), Maxwell 16 Tissue DNA Purification Kit (Promega Biotech AB, Stockholm, Sweden), DNeasy Blood & Tissue Kit (Qiagen), QIAamp Fast DNA Stool Mini Kit (Qiagen), QIAamp DNA Stool Mini Kit (Qiagen) and QIAamp cador Pathogen Kit (Qiagen). Each kit was tested following the provided kit manuals, with a few exceptions (Table 1). If applicable, extractions started with a pretreatment consisting of a heat shock step followed by bead beating treatment. The pretreated samples then entered the extraction process and the eluates from the extractions were finally evaluated quantitatively by NanoDrop and/or Qubit, as well as qualitatively by agarose gel electrophoresis and/or performance in PCR. First, six different DNA extraction kits were tested in an initial investigation. The extraction kit yielding the highest DNA concentration together with the QIAamp Stool DNA kit (as a reference) was further investigated, since the latter is the most commonly used kit for fecal DNA extraction from Antarctic birds [34,38–40]. Finally, the extraction kit yielding the highest DNA concentration was optimized for DNA extraction from feces of Antarctic bird species.

**Table 1.** Modifications of the different DNA extraction kits tried in the current study.

ID	Kit Name	Loaded sample amount	Heat shock	Bead beating	Volume supernatant used	Sample source	Approximate completion time	Additional comment
E1	PowerSoil DNA Isolation Kit	250 mg	No	2 × 5 min	1900 μL	Mallard	40 min	
E2	Maxwell 16 Tissue DNA Purification Kit	50–100 mg	“	No	N/A	“	45 min	Solid feces put directly into the kit
E3	“	50–100 mg	Yes	2 × 5 min	800 μL	“	70 min	Solid feces dissolved in 1 mL 1 × PBS
E4	DNeasy Blood & Tissue kit	150 mg	“	1 × 5 min	800 μL	“	160 min	Solid feces dissolved in 800 μL 1 × PBS Proteinase K treatment 56°C 70 min
E5	“	200 mg	“	3 × 20 s	200 μL	“	“	Feces in LB glycerol
E6	QIAamp Fast DNA Stool Mini Kit	200 mg	No	No	200 μL	Mallard	45 min	Solid feces
E7	“	“	Yes	3 × 20 s	“	Mallard	“	Feces in LB glycerol
E8	QIAamp DNA Stool Mini Kit	200 mg	Yes	3 × 20 s	“	Mallard	65 min	Feces in LB glycerol
E9	“	“	“	“	“	“	“	Solid feces
E10	“	“	“	2 × 5 min	“	“	70 min	Solid feces
E11	QIAamp cador Pathogen	“	“	3 × 20 s	“	“	45 min	Solid feces dissolved in 500 μL 1 × PBS Proteinase K 70°C 10 min
E12	“	“	“	“	“	“	“	Solid feces dissolved in 500 μL ASL Proteinase K 70°C 10 min
E13	“	“	“	“	“	Penguin	“	Solid feces dissolved in 800 μL ASL Proteinase K 70°C 10 min
E14	“	150 mg	“	“	“	Gull	“	Solid feces dissolved in 800 μL ASL Proteinase K 70°C 10 min
E15	“	100 mg	“	“	“	Gull; Penguin	“	Solid feces dissolved 1 mL ASL Proteinase K 70°C 10 min

The sign ““ denotes that the value/setting was identical to the one given directly above.

### Sample source and type

Fresh fecal droppings from the following species were used in this study: mallard (*Anas platyrhynchos*), gentoo penguin (*Pygoscelis papua*), Adélie penguin (*Pygoscelis adeliae*), chinstrap penguin (*Pygoscelis antarcticus*), snowy sheathbill (*Chionis albus*), kelp gull (*Larus dominicanus*) and brown skua (*Catharacta antarctica*). Feces from penguins, sheathbills, gulls and skuas were collected as fresh droppings from wild individuals at the Antarctic Peninsula, Antarctica. The samples were collected using sterile cotton swabs (Sarstedt AB, Helsingborg, Sweden) and stored dry in 2 mL screw cap microtubes (Sarstedt AB) without the cotton swab at −80°C. The mallards were captive, kept for research purposes and fed with commercial duck feed, Penna (Lantmännen Lantbruk, Malmö, Sweden) from Day 1 to 6 weeks of age and then fed with Plym (Lantmännen Lantbruk, Malmö, Sweden) until euthanasia. The mallard fecal samples were collected using sterile cotton swabs (Nordic Biolabs AB, Täby, Sweden) and stored either dry or in LB glycerol (Dept of Clinical Microbiology, Uppsala University, Uppsala, Sweden) in 2 mL screw cap microtubes (Sarstedt AB, Helsingborg, Sweden) at −80°C. Mallard feces were used in the initial investigation. Mallard and Antarctic avian feces were used in the further investigation.

Mallard feces are highly fibrous and of semiliquid to solid state. Penguin feces are of a semisolid state with a very high content of non-digested crustacean shells (mainly from krill, *Euphausiacea*). Sheathbill and skua feces are similar to penguin feces, but skua feces contain residuals of a more opportunistic diet including feathers and bones from other birds. Gull feces are of more liquid state, but also comprise residuals of an opportunistic omnivorous diet.

### Pretreatment

Samples were thawed on ice for a minimum of 60 min. Depending on the physical state (either solid feces or LB glycerol liquid dispersion), an aliquot of feces was separated by a sterile instrument, or the liquid dispersion was vortexed for 1 min and centrifuged at 500 *g* for 1 min and a liquid aliquot was withdrawn from the supernatant (Table 1). When applicable, the sample aliquot was heat shocked by incubation at 95°C for 5 min followed by incubation on ice for 5 min. After the heat shock, the sample was instantly bead-beaten at 5000 rpm in a Bio 101 FastPrep FP120-120V disrupter homogenizer bead beater (Savant, Illkirch-Graffenstaden, France). Bead beating varied from 3 × 20 s, 2 × 50 s, 1 × 5 min and 2 × 5 min with 300 mg of 0.1 mm silica beads cat. no. 11079101z (BioSpec Products, Bartlesville, OK, USA) per tube. The samples were kept on ice and during repeated bead beatings, the samples were incubated on ice for 1 min between each bead-beating repeat. Beads were pelleted by centrifugation at 2500 *g* for 1 min. An aliquot of the supernatant was then loaded to the DNA extraction kit; some of the DNA extraction kits also contained a proteinase K treatment (Table 1).

### DNA quantification and quality control

The DNA content of the eluates from each extraction method was evaluated by at least one of the following methods: NanoDrop2000c (Thermo Fisher Scientific, Waltham, MA, USA), Qubit 1.0 fluorometer (Thermo Fisher Scientific) with the Qubit dsDNA HS Assay Kit (Thermo Fisher Scientific) and agarose gel electrophoresis, 0.5–0.8% agarose (VWR Chemicals, Spånga, Sweden) in 1 × TAE buffer (Sigma-Aldrich AB, Stockholm, Sweden). During the initial investigation DNA yields were evaluated using NanoDrop2000c for rapid measurements. In the further investigation of kit performance, NanoDrop measurements were complemented by Qubit measurements. A subset of extracts was selected for evaluation in PCR with two different setups. All Antarctic samples’ eluates (extracted by the QIAamp cador Pathogen kit) were tested in a 16S rDNA PCR due to the complex nature of these avian species’ feces. Mallard fecal extracts from the QIAamp Fast DNA Stool, QIAamp DNA Stool and QIAamp cador Pathogen kits were tested in 16S rDNA PCR or a PCR specific for the bacterium *Campylobacter jejuni*. The primer sequences, PCR reagents and thermal cycling conditions of the 16S rDNA PCR are presented in supplementary material S1. In the 16S rDNA PCR, the templates were diluted 1:10, 1:100 and 1:1000 to reduce the probability of PCR inhibition. The *Campylobacter jejuni* specific primers were targeting part of the *glnA* gene. The *Campylobacter* specific PCR is described in supplementary material S2. In the *Campylobacter* PCR, the templates were added undiluted and 1:10 and 1:100 times diluted. All mallard fecal samples in LB glycerol were tested both in the conventional *Campylobacter* PCR and later in a real-time PCR. The development of the real-time PCR is described elsewhere (Atterby *et al*. unpublished observations).

## Results

### Initial investigation of six different DNA extraction kits

**Figure 1.**
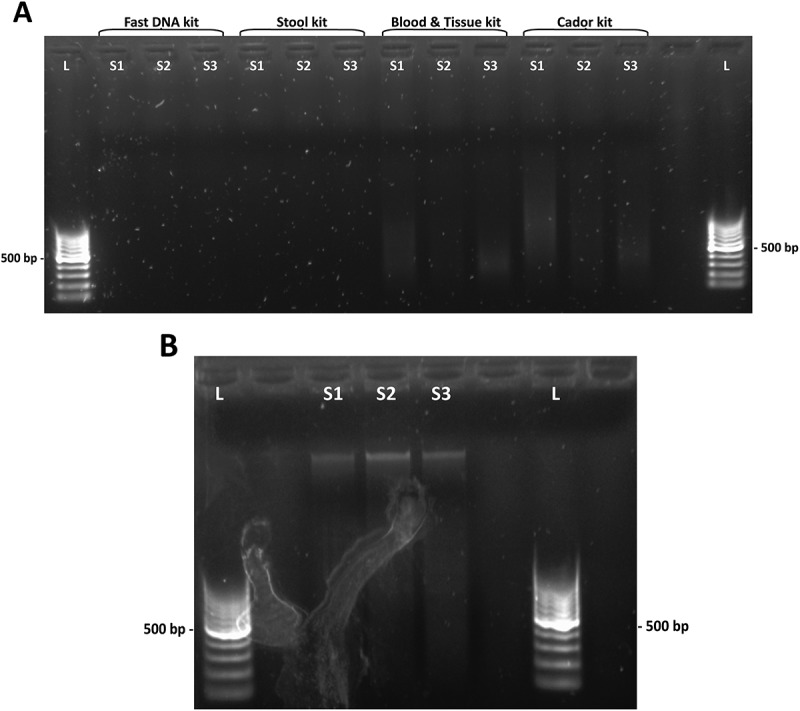
Agarose gel electrophoresis of kit eluates. (a) DNA yields after extraction with four different kits. From left: QIAamp Fast DNA Stool Mini Kit, QIAamp DNA Stool Mini Kit, DNeasy Blood & Tissue Kit and QIAamp cador Pathogen Kit. Faint smears observed in the DNeasy Blood & Tissue kit and the QIAamp cador Pathogen kit lanes. (b) DNA yields after bead beating pretreatment and extraction with QIAamp cador Pathogen kit. *L*, DNA ladder. *S1, S2* and *S3*, fecal extracts.

In an initial investigation, six different DNA extraction kits were tested for fecal DNA extraction using feces from mallards. The complex matrix of mallard feces made DNA extraction challenging with very poor DNA yield, indicating the need of pretreatment and/or optimization, see Figure 1(a). In general, it was observed that the less liquid state of the fecal sample, the more difficult to extract DNA from it. Indeed, mallard feces were the most simple to extract DNA from, whereas penguin and sheathbill feces were the most challenging.

**Figure 2.**
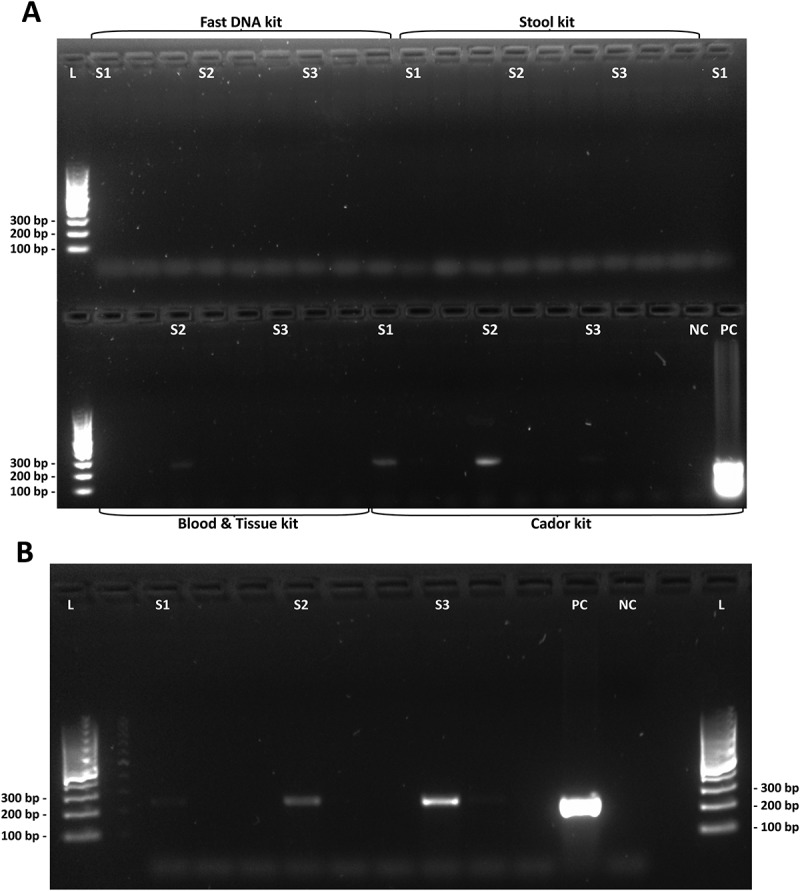
Agarose gel electrophoresis of *C. jejuni*-specific PCR with mallard eluates. (a) PCR products from DNA samples extracted with four different kits. Top row from left: QIAamp Fast DNA Stool Mini Kit, QIAamp DNA Stool Mini Kit and DNeasy Blood & Tissue Kit (sample S1). Bottom row from left: DNeasy Blood & Tissue Kit (samples S2 and S3) and QIAamp cador Pathogen Kit. Faint bands were detected in samples extracted with DNeasy Blood & Tissue kit and QIAamp cador Pathogen kit (bottom row). (b) Intense bands visible in samples pretreated with combined heat-shock and bead beating and extracted with the QIAamp cador Pathogen kit. *L*, DNA ladder. *S1, S2* and *S3*, consecutive samples. *NC*, negative control. *PC*, positive control.

The PowerSoil DNA Isolation kit from MO Bio (MO BIO Laboratories Inc., Carlsbad, CA, USA) was tested, but gave a very low eluate DNA concentration (Table 2). An automated robotic extraction kit (Maxwell 16 Tissue DNA Purification kit) from Promega (Promega Biotech AB, Stockholm, Sweden) was tested according to the manufacturer´s instructions, as well as with pretreatment. When the kit was used without pretreatment, the eluted extracts had a very high content of carryover beads from the extraction, which made accurate quantification difficult. However, when this kit was combined with heat shock and bead beating, the DNA yield was low. The yield from the DNeasy Blood & Tissue kit was low to moderate, performing better with liquid dispersion of fecal samples and producing a faint band from a mallard fecal extract when tested with the conventional PCR specific for *C. jejuni* (Figures 1(a) and 2(a)). The QIAamp Fast DNA Stool Mini kit did not yield any DNA (Table 2, Figures 1(a) and 2(a)). The QIAamp cador Pathogen Mini kit performed best in the initial investigation (Table 2).

**Table 2.** The outcome of the different DNA extraction kits and their alterations tested in the current study.

ID	Kit name	NanoDrop ng/μL	A260/A280	A260/A230	QC gel electrophoresis	16S PCR gel
E1	PowerSoil DNA Isolation Kit	5.70	2.08	0.685	Empty	N/A
E2^a^	Maxwell 16 Tissue DNA Purification Kit	163^a^	0.695	0.200	Faint smear high-weight fragments	“
E3	“	6.30	3.31	0.990	Faint smear middle-weight fragments	“
E4	DNeasy Blood & Tissue Kit	4.75	0.625	0.840	Empty	“
E5	“	16.0	1.51	0.175	Faint smear middle-weight fragments	“
E6	QIAamp Fast DNA Stool Mini Kit	0	N/A	N/A	Empty	Empty
E7	“	1	“	“	“	“
E8	QIAamp DNA Stool Mini Kit	1	“	“	“	“
E9	“	9.90	0.475	0.4825	“	N/A
E10	“	4.35	2.12	0.940	“	“
E11	QIAamp cador Pathogen Kit	10.3	4.82	0.460	Faint smear high-weight fragments	30 cycles faint bands 10^0–(−2)^ dilution
E12	“	75.3	1.20	0.815	Strong smear high-weight fragments	30 cycles strong bands 10^(−1)–(−2)^ dilution
E13	“	17.4	1.58	0.385	N/A	35 cycles 10^(−2)^ dilution weak bands, 10^(−3)^ dilution strong bands
E14	“	77.1	0.795	0.350	“	35 cycles 10^(−2)^ dilution strong bands, 10^(−3)^ dilution weak bands
E15	“	44.1; 8.40	1.63; 1.67	0.810; 1.10	“	“

^a^Very heavy bead carryover interfering with eluate quantification.

The sign " denotes that the value/setting was identical to the one given directly above.

### Further investigation of optimal extraction methodology

Because the QIAamp cador Pathogen Mini kit gave the highest DNA yields, it was decided to continue to investigate the effect of further optimization attempts on this kit (Figure 1). The QIAamp DNA Stool Mini kit was included as reference, since this kit earlier has been used for avian fecal DNA extraction [40]. However, the yield from the QIAamp DNA Stool Mini kit was low, even in combination with heat shock and bead beating (Table 2). When applying the same pretreatment before extraction of mallard solid feces with each of the two kits, the QIAamp DNA Stool Mini kit gave an eluate concentration of 9.90 ng/μL in contrast to 75.3 ng/μL for the QIAamp cador Pathogen kit, as measured by NanoDrop. The QIAamp cador Pathogen kit in combination with heat shock and bead beating was the most successful method tried, yielding a higher eluate DNA concentration than any of the other extraction kits in the study (Table 2, Figure 2). Thus, it was decided to make the final optimization based on the QIAamp cador Pathogen kit. The modifications of the kit improved the DNA yield (Figure 1(b)), compared to the unmodified kit protocol and gave better-quality detection bands after the conventional PCR specific for *C. jejuni* (Figure 2(b)).

A final extraction protocol based on the QIAamp cador Pathogen kit was formulated (Table 3). With the optimization procedure applied, it was possible to increase the DNA yield from solid mallard feces from a few ng/μL to 75.3 ng/μL and from solid penguin feces from zero to 14.7 ng/μL (mean of 40 individual penguin fecal samples). Fecal samples from the Antarctic bird species of interest in the current study, as well as mallard fecal samples, were processed according to the protocol described in Table 3. The NanoDrop values were ~10–20 times higher than Qubit values (Table 4). The concentration of the eluted extracts on average, ranged from ~14–75 ng/μL as measured by NanoDrop. Mallard feces yielded the most DNA, whereas penguins’ and sheathbills’ samples yielded the least DNA. Bead beating and vortexing were required to fragment the semisolid feces, but extensive bead beating also reduced the extracted DNA integrity (data not shown). When larger sample amounts were loaded to the spin columns the yield decreased, indicating saturation of the spin columns. The optimum sample amount for this set of samples was in the range of 50–150 mg sample per spin column.

**Table 3.** Suggested pretreatment and extraction protocol.

Step	Time
Thaw sample on ice	>1 h
300 mg 0.1 mm silica beads/tube (BioSpec Products Cat# 110 79101)	
100–150 mg sample/tube	
1 mL ASL stool lysis buffer/tube (Qiagen Cat# 19082)	
Vortex	1 min
Heat shock 95°C	5 min
Heat shock incubation on ice	5 min
Bead beating 5000 rpm	3 × 20 s
Incubation on ice between each bead beating set	1 min
Pellet beads by centrifugation at 2500 *g*	1 min
Load 200 μL supernatant/sample into the QIAamp cador Pathogen Kit	
Follow the QIAamp cador Pathogen Kit Protocol with the following adjustments:	
● Proteinase K treatment at 70°C	10 min
● Elute the extract in 100 μL of the included AVE buffer/tube

**Table 4.** DNA yields from avian fecal DNA extractions following the protocol presented in Table 3 from fecal samples obtained from seven bird species.

	Mean ng/μL		
	NanoDrop	Qubit	Number of samples
Mallard	75.4	N/A	6
Gentoo penguin	14.5	0.880	16
Adélie penguin	13.9	1.00	13
Chinstrap penguin	15.7	2.55	11
Snowy sheathbill	15.5	1.03	10
Kelp gull	26.4	2.91	8
Brown skua	20.8	1.42	3

## Discussion

Culture-independent analysis techniques have become very important tools to study microbial communities, but these techniques are highly dependent on an efficient DNA extraction procedure [20]. The complexity of feces requires optimization to reach an efficient DNA extraction with a high-quality output for downstream applications. A fundamental cause of the issues of DNA extraction from avian feces is the nature of birds mixing digestive residuals and urinary compounds to a single heterogeneous fecal deposit [32]. This can result in a cocktail of molecules that interfere with the extraction, including uric acid, bile salts, nucleases and partly/non-degraded complex polysaccharides [20,38,41].

In the initial investigation, six different DNA extraction kits were evaluated using mallard fecal samples. When extraction procedures followed the kit manufacturers’ protocols, the eluted DNA yields were very poor or absent (Table 2, Figure 1(a)). Even the so-called ‘feces-specific’ DNA extraction kits performed very poorly. Indeed, these kits were among the least successful. Extracts from the initial investigation were also tested in a *Campylobacter-*specific PCR where the QIAamp cador Pathogen kit eluates showed the best results (Figure 2(a)). The automated DNA extraction by the Maxwell robot from Promega suffered from a very high bead carryover, interfering with downstream applications.

The QIAamp cador Pathogen kit was selected for further investigation, since it performed the best in the initial investigation (Figure 1(a)) and the QIAamp DNA stool kit was used as reference. Bead beating was required to extract DNA from this sample set, yet bead beating may be destructive to the DNA quality and too extensive bead beating should be avoided, since impaired DNA integrity may interfere with downstream applications [42]. A repeated set of bead beatings were shown to reduce DNA shear compared to one long bead-beating step (data not shown). When applying bead beating, the efficiency of the DNA extraction improved greatly for the QIAamp cador Pathogen kit eluting high M_w_ DNA (Figure 1(b)). However, the QIAamp DNA Stool Mini kit did not reach the performance of the QIAamp cador Pathogen kit despite applying the same pretreatment (see Table 2 and Figure 1(b)). The difference in performance of the QIAamp cador Pathogen and the QIAamp DNA Stool Mini extraction kits addresses the difference in the nature of avian and human feces and that different types of feces require different extraction protocols for successful DNA extraction.

In the initial investigation, six different DNA extraction kits were evaluated using duplicate mallard feces samples and therefore no statistical evaluation could be made. Yet, in all comparisons the QIAamp cador Pathogen DNA extraction kit consistently yielded the most DNA. Indeed, in most cases the other DNA extraction kits failed to yield any eluted DNA. After final optimization, a larger set of samples was extracted according to the protocol presented in Table 3 and good DNA yields were obtained from all avian species (Table 4). A noteworthy difference in DNA yields was observed between avian species. This can probably be explained by the different composition of the fecal deposits from the different bird species. The mallard feces are fibrous and semiliquid, whereas the penguin feces used were almost completely solid. The vast majority of penguin feces consists of crustacean shells from krill in the penguin diet. The krill shells give rise to an extraordinary high level of chitin-like substances, which challenged DNA extraction. The observed difference in DNA yield depending on the host species highlights the importance of the sample matrix composition and heterogeneity of avian feces. Fecal DNA extraction from other avian orders might require further optimization of the presented method, to make it fully compatible with the avian species of interest.

The dual nature of avian deposits – being a mixture of fecal material and urinary deposits, including uric acid – further challenges DNA extraction. Uric acid is not only antagonistic to the DNA extraction itself, but complicates direct spectrophotometric quantification of extracted DNA, since uric acid has an absorbance maximum at 292 nm that may interfere with DNA quantification [43]. The NanoDrop is a rapid quantification method, but compared to the Qubit dsDNA High Sensitivity kit, the NanoDrop readings were 10–20 times higher. Since the Qubit dsDNA High Sensitivity kit is the most accurate DNA quantification technique employed, one should be aware of the overestimation of DNA concentration made by the NanoDrop. However, the NanoDrop provides estimates of the purity of DNA towards proteins and residual extraction chemicals that is of interest during an optimization procedure.

A 1:100 dilution of the solid-feces DNA extracts resulted in the best PCR performance, probably because bird feces contains many PCR inhibitors [41]. Extracts from mallard feces in LB glycerol performed the best undiluted in the PCR, but adding the solid feces to LB glycerol and consecutively adding the sample supernatant to the lysis buffer generated an overall dilution factor of ~1/100. However, the PCR sensitivity to inhibitors in the extracts depends on many factors, including what PCR kit/reagents are used [20].

The extraction protocol presented in Table 3 was not investigated for potential extraction bias toward any specific group of organisms. However, fecal samples from mallards experimentally infected with *C. jejuni* and extracted according to the protocol in Table 3 were further analyzed with a quantitative real-time PCR for quantification of fecal bacterial numbers. The qPCR data were validated against estimates of bacterial numbers by plate counts with good concordance, indicating that this extraction method is suitable for such analysis (Atterby *et al*. unpublished observations). Similarly, DNA was successfully extracted from a collection of feces from Antarctic birds following the procedure presented in Table 3. These eluates were then used for NGS library preparation and analyzed in a microbiota project (Eriksson *et al*. unpublished observations).

These results show that the optimized protocol based on the QIAamp cador Pathogen kit can be used for extraction of DNA from bird feces for microbiota analysis where DNA is ideally extracted from as many microbial species as possible, as well as for infection experiments where usually one bacterial pathogen is targeted. In this study, *C. jejuni* was used and the optimized extraction method yielded high-quality DNA both for bacterial detection by endpoint PCR and accurate bacterial quantification using qPCR. The majority of earlier studies describing birds as models for *C. jejuni* infection experiments have quantified the bacteria by plate count [18,44–46]. In contrast, fewer studies have utilized culture-independent techniques to detect and quantify the bacterium [14,47]. Non-optimal DNA extraction might be a reason why some studies have not detected the bacterium in fecal samples where its concentration might have been low [48].

In conclusion, the performance of six DNA extraction kits was compared, when applied to feces from different bird species. After initial evaluation of DNA yields of the extracts, the QIAamp cador Pathogen kit performed best and was selected for further optimization. The study provides an optimized protocol, which proved to yield high-quality DNA from feces of seven different bird species of different avian orders. It should be noted that the QIAamp cador Pathogen kit was selected after extractions performed according to the standard protocols for each kit tested. Hence, it cannot be excluded that any of the other DNA extraction kits might yield high-quality DNA from the same bird fecal samples after optimization of their respective protocols. The current study presents an approach to DNA extraction from ‘difficult to extract’ avian feces that might be applicable to other difficult samples of various sources.

## Supplementary Material

Supplemental Data
