# Supplementary material for: Evaluation and optimization of microbial DNA extraction from fecal samples of wild Antarctic bird species
Source: Infect Ecol Epidemiol. 2017 Oct 26;7(1):1386536. doi: 10.1080/20008686.2017.1386536 (PMC5678435; doi:10.1080/20008686.2017.1386536)
Supplement: Supplemental Data [file ZIEE_A_1386536_SM6775.docx]

**Supplements**

**S1. Conventional 16S PCR**

The primers targeting the 16S rDNA were: forward 5’-GTG BCA GCM GCC GCG GTA-3’ and reverse 5’-GAC TAC HVG GGT ATC TAA TCC-3’. All reagents were purchased from Applied Biosystems (Thermo Fischer Scientific, Waltham, MA, U.S.A.). The composition at the start of the PCR was the following: 1×PCR Gold Buffer, 200 μM dNTPs, 2.5 mM MgCl_2_, 0.5 μM 515 F, 0.5 μM 805 R and 1.25 U AmpliTaq. The temperature profile of the PCR was the following: 95 °C for 10 min, 35×(98 °C for 30 s, 59 °C for 30 s, 72 °C for 1 min 15 s) and 72 °C for 10 min.

**S2. Conventional PCR specific for *C. jejuni***

The *Campylobacter* specific primers are listed in table S3. The forward and reverse primers amplifying either the glutamine synthetase (glnA) or the phosphoglucomutase (pgm) alleles are shown. The sequence type (ST) that the alleles belong to is also shown in the table.

Table S3 *Campylobacter* specific primers.

| Sequence Type | Target gene | Primers | (5’ 🡪 3’) |
| --- | --- | --- | --- |
| ST–104 & ST-995 | glnA | ACCGAAGAAGGAGAGTGGAA | Forward |
| ST-104 & ST-995 | glnA | GCTTCTACAAGCGTGCCAA | Reverse |
| ST-104 & ST-995 | pgm | TGATGGTGATGCAGATCGTT | Forward |
| ST-104 & ST-995 | pgm | TCCACCAAAATTTCCACCAT | Reverse |

Each PCR reaction consisted of 1 x AmpliTaq Gold PCR buffer (Thermo Fischer Scientific, Waltham, MA, U.S.A.), 2.5 mM MgCl_2_ (Thermo Fischer Scientific, Waltham, MA, U.S.A.), 200 μM Fermentas dNTPs (Thermo Fischer Scientific, Waltham, MA, U.S.A.), 1.25 units of AmpliTaq Gold ^®^ DNA Polymerase (Thermo Fischer Scientific, Waltham, MA, U.S.A.), 0,2 µM of each of the primers (table X), 10 ng of genomic DNA and DEPC treated water from Invitrogen (Thermo Fischer Scientific, Waltham, MA, U.S.A.) to a final volume of 25 μL. The PCR conditions used were followed as in Denis S. et al., (1999). PCR amplification was performed using the following conditions: 95 ^o^C for 10 min, followed by 25 cycles consisting of 95 ^o^C for 30 sec, 63 ^o^C for 90 sec, 72 ^o^C for 1 min and a final extension step of 72 ^o^C for 10 min. PCR reactions were performed using Bio-Rad CFX96 Optics Module C1000 Thermal Cycler (Bio Rad Laboaratories AB, Sundbyberg, Sweden) and 2720 Therma Cycler (Thermo Fischer Scientific, Waltham, MA, U.S.A.).
